# A good beginning: study protocol for a group-randomized trial to investigate the effects of sit-to-stand desks on academic performance and sedentary time in primary education

**DOI:** 10.1186/s12889-019-8135-9

**Published:** 2020-01-15

**Authors:** A. (Lex) E. Q. van Delden, Guido P. H. Band, Joris P. J. Slaets

**Affiliations:** 1 Leyden Academy on Vitality and Ageing, Leiden, The Netherlands; 2 Department of Public Health and Primary Care, Leiden University Medical Center, Leiden, The Netherlands; 3 Cognitive Psychology Unit, Institute of Psychology, Leiden University, Leiden, The Netherlands; 4 Leiden Institute for Brain and Cognition (LIBC), Leiden, The Netherlands; 5 Center for Geriatric Medicine, University Medical Center Groningen, Groningen, The Netherlands

**Keywords:** Sedentary behavior, Primary school, Children, Health, Academic performance, Cognition, Quality of life, Intervention, Sit-to-stand desks

## Abstract

**Background:**

Sedentary behavior is associated with health risks and academic under-achievement in children. Still, children spend a large part of their waking hours sitting at a desk at school. Recent short-term studies demonstrated the potential of sit-to-stand desks to reduce sitting time in primary education. The program of “A Good Beginning” was conceived to assess the long-term effects of sit-to-stand desks on sitting time in primary education, and to examine how sit-to-stand desks versus regular desks relate to academic performance, and measures of executive functioning, health and wellbeing. The present paper describes the design of this group-randomized trial, which started in 2017 and will be completed in 2019.

**Methods:**

Children of two grade-three groups (age 8–9) following regular primary education in Leiden, The Netherlands, were recruited. A coin toss determined which group is the experimental group; the other group is the control group. All children in the experimental group received sit-to-stand desks. They are invited and motivated to reduce sedentary time at school, however, it is their own choice to sit or stand. Children in the control group use regular desks. Otherwise, both groups receive regular treatment. Outcomes are assessed at baseline (T0) and at five follow-up sessions (T1-T5) alternately in winter and summer seasons over three academic years. Primary outcome measures are academic performance, and the proportion of sitting time at school, measured with a 3D accelerometer. Secondary outcome measures are a number of measures related to executive functioning (e.g., N-back task for working memory), health (e.g., height and weight for BMI), and wellbeing (e.g., KIDSCREEN-52 for Quality of Life).

**Discussion:**

A Good Beginning is a two-and-a-half-year research program, which aims to provide a better understanding of the long-term effects of sit-to-stand desks on sedentary time at school and the relation between sitting time reduction and academic performance, executive functioning, health and wellbeing. The findings may serve as useful information for policy making and practical decision making for school and classroom environments.

**Trial registration:**

The program of “A Good Beginning” is registered at the Netherlands Trial Register (NTR, https://www.trialregister.nl), number NL6166, registration date 24 November 2016.

## Background

Following the advances in technology in the past century, people nowadays spend the largest part of their time awake in a sitting position [1, 2]. In adults, sedentary behavior is associated with serious health risks, such as obesity, cardio-vascular diseases, diabetes, and a reduced cardio-respiratory fitness [3]. Although less consistent than in adults, sedentary behavior is also related to health risks in children [4, 5]. In addition, children’s sedentary behavior is associated with academic under-achievement [4, 5].

Children spend a large part of their waking hours at school sitting [6–8], in a classroom that is designed for sitting. Children’s sedentary behavior, implicitly learned in school, may continue into adolescence [7–11] and adulthood [9, 10, 12]. Consequently, from childhood onwards, sedentary behavior becomes the rule, rather than the exception. Therefore, the school setting, in particular, is the place to reduce children’s sedentary time and promote standing and active behavior, which may also prevent excessive sedentary behavior in later age. Given the fact that the environment has a very strong influence on behavior [13–15], sit-to-stand desks have the potential to invite and seduce children to sit less and to promote standing and active behavior.

On the one hand, reduced sitting time [16–21] and increased energy expenditure [22–24] are relevant physical benefits of using desks that promote standing found in previous studies. On the other hand, concerns about the use of such desks have been expressed by teachers and the long term impact on academic performance is unclear [25]. In short term studies, academic performance didn’t seem to suffer [26]. Moreover, better working memory capabilities [24], and attention and task focus [27] (i.e., the mechanisms suggested to underlie the relation between executive function and academic performance [28]) are advantages that come with the use of desks that promote standing. Hence, academic performance (and cognitive skills) may not suffer from, and even improve with, less sitting and more standing. However, all these benefits have only been reported in short-term studies with a duration of a year or (in most studies, much) less, which may suffer from effects of novelty and season. Students’ enthusiasm to sit less may wane when the novelty of the sit-to-stand desks wears off (cf. [29]), and children are more activity prone in summer than in winter [30].

The program of “A Good Beginning” entails a group-randomized trial in which the merits of sit-to-stand desks in the primary school classroom are investigated over a two-and-a-half-year period, beyond the effect of novelty and controlling for season. To this end, two grade-three groups (students aged 8–9 years) were recruited. In The Netherlands, grade-three students are the oldest students to recruit for a study of this duration. In the final year of primary school, sixth grade, the curricular activities differ notably from the activities in the curriculum of grades one to five. Children in one group, the experimental group, received sit-to-stand desks for the entire study. Students in the control group use regular, seated desks. The results of the program are expected by the beginning of 2020.

The primary aim of the program of “A Good Beginning” is to assess possible harm inflicted on academic performance as an adverse event of long-term implementation of sit-to-stand desks in the primary school classroom. Based on previous findings in shorter term studies [24–27, 31], we expect that the sit-to-stand desks are proper alternatives to regular, seated desks without negative effects on academic performance, and possibly with positive effects. Secondly, in terms of effectiveness, the program aims to assess the long-term effect of sit-to-stand desks on sedentary time. Based on earlier findings, a reduction in sedentary time may be expected.

Furthermore, to gain a broader view on the long term effects, cognitive skills and indicators of health and wellbeing are investigated in relation to sedentary time. Cognitive skills relevant to academic performance, such as working memory, planning, inhibition, and cognitive flexibility, may be influenced by sedentary time [24, 27, 28]. Inactivity and sedentary behavior are negatively associated with wellbeing [32–34], physical fitness [4, 5], and strength [4], and positively associated with childhood obesity [4, 5], (cf. [35]), constipation [36, 37], and a higher risk of insomnia and sleep disturbance [4, 38]. Moreover, short sleep duration and sedentary behavior together are associated with childhood obesity [39], while executive function appears to mediate between sleep duration and sedentary behavior [40].

The outcomes of this study will provide a better understanding of the effects of the classroom environment on academic performance, sedentary time, cognition, health, and wellbeing. The findings may serve as useful information for policy making and practical decision making with regard to school and classroom environments, as well as for future long term efficacy trials on a larger scale. With this study protocol, together with the ethical approval and trial registration, we wish to contribute to transparency, reduce publication bias, and improve reproducibility. This study protocol prevents unnecessary duplication of research, and indicates when to expect the results and findings.

## Methods

The program of “A Good Beginning” has received ethical approval from the Dutch Central Committee on Research Involving Human Subjects (CCMO, https://english.ccmo.nl, number NL60159.000.17). This study is conducted according to the principles of the Declaration of Helsinki [41] and in accordance with the Medical Research Involving Human Subjects Act (WMO) [42]. Risks associated with participation and physical and psychological discomfort are very small to negligible. There are no risks related to the use of sit-stand desks other than, or additional to, the use of regular desks. A SPIRIT checklist covering all recommended trial protocol items is provided as Additional file 1.

### Design

**Fig. 1:**
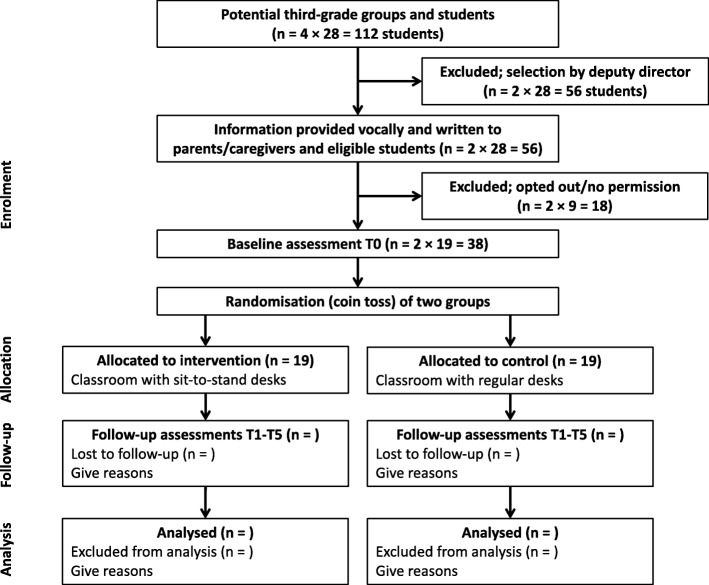
Flow-chart of the program of “A Good Beginning”

This is a prospective, two-armed, group-randomized trial (see Fig. 1). The trial started in 2017 and will be completed in 2019. Outcomes are assessed at baseline (T0; May 2017) and at five follow-up sessions (T1-T5) with an interval of approximately 6 months, alternately in summer (i.e., July 2017, 2018 and 2019) and winter (i.e., January/February 2018 and 2019) seasons over three academic years. After the baseline assessment, which took place after informed consent, a coin toss (by AEQ in the presence of the teachers and the deputy director) determined which group is the experimental group; the other group is the control group. Most tests during the assessment periods take place at school; activity tracking and keeping diaries (also) take place outside the school.

Blinding is not possible for children, teachers and parents/caregivers. Attempts are taken to keep assessors blinded. However, working with children in this age range proves to be difficult to uphold blindness of assessors. Nevertheless, the influence of assessors on most, and at least on the primary outcomes is considered minimal. The assessors are neither present at the time of measuring sitting time nor at testing academic performance. The assessors are trained junior researchers from Leyden Academy on Vitality and Ageing (Leyden Academy) and the Cognitive Psychology Unit from the Institute of Psychology at Leiden University. Under primary and secondary outcome variables we indicate for which outcome assessment the assessors (and teachers) are present for supervision.

Each year the study is evaluated by the principal investigators, the involved teachers, the school (deputy) director, and members of the Parent-Teacher Association. During this meeting, the progress of the study, as well as safety issues related to testing and the use of the sit-stand desks are discussed.

### Recruitment

Students of two grade-three groups (aged 8–9 years) following regular primary education were recruited from a school in Leiden, The Netherlands, of which the school director initiated this study. At this school there were four grade-three groups. The common group size is 26 to 30 students. The deputy director of the school selected two groups in consultation with the teachers, based on the willingness of the teachers to be involved in the study. All children in both groups were asked to participate. The allocation was only established after baseline assessment (which was after informed consent). In order to be eligible to participate in this study, a student had to meet all of the following criteria:

- Follow regular primary education in third grade
- Have a signed Informed Consent form to participate; given the age, parents/caregivers should sign the Informed Consent form for their child but the student will be asked to sign one as well
- Be physically able to stand without any serious health issues or injuries; note that a student who is normally physically able to stand, but temporarily unable to do so because of a recovery from a temporary injury of trauma, can still participate in this study

Students who object to participate are not included in the study. Examples of objection are (signals of) fear, sadness, and anger. We expected to recruit around 20 students per group. Students who did not participate from the start of the study, but wish to do so at a later moment, are also enrolled at the next moment of testing. Those who terminate their participation before the end of the study are asked for the reason(s) why. Demographics of students that in fact are recruited (and for whom we received written informed consent from themselves and their parents/caregivers) are presented in Table 1.

**Table 1:** Participants’ age and sex

	Control group	Intervention group	Total
	(n = 19)	(*n* = 19)	(*n* = 38)
Years of age median [range]	9.0 [8.5 to 10.2]	8.9 [8.5 to 10.3]	8.9 [8.5 to 10.3]
Girls n (%)	10 (52.6)	11 (57.9)	21 (55.3)

### Sample size, power, and non-inferiority

In relation to possible harm to academic performance as an adverse event of long term use of sit-to-stand desks, we will investigate (non-)inferiority, rather than the effectiveness of sit-to-stand desks on measures of academic performance. For this, the 95% confidence interval (CI) and a margin of non-inferiority (the maximum acceptable extent of non-inferiority of an experimental treatment) are relevant. For the following calculations we have used the expected 20 students per group, and the distribution data of academic performance provided by CITO [https://www.cito.com]. Note that the data of the school participating in this study are similar to the data provided by CITO:

$$ \mathrm{We}\ \mathrm{assume}:{\mathrm{n}}_{\mathrm{exp}}={\mathrm{n}}_{\mathrm{con}}=20\ \mathrm{and}\ \mathrm{equal}\ \mathrm{variance} $$

**Table:** 

	Reading comprehension	Arithmetic	Orthography
Mean	159	214	214
SD	25	26	26
SE diff	7.9	8.3	8.3
95% CI diff	15.8	16.6	16.6
% mean	10	7.8	7.8

This means that for accepting non-inferiority in case μ = 0 or μ > 0, at maximum a difference between the mean scores of both groups of around 10% will be accepted. If μ < 0 (i.e., the mean score of the experimental group is more than 10% lower than the mean score of the control group), non-inferiority will not be accepted.

With regard to the proportion of sitting time (i.e., second primary outcome), we look at the effectiveness of sit-to-stand desks. For these calculations we used the results from the study by Clemes et al. [17]. We used the pooled statistics of the follow-up assessment of the control groups in this study to calculate the 95% CI. Based on these results, chances are small that in this study, with an expected 20 participants in each group, a significant difference in sitting time will be found between groups at the end of the study (i.e., the α-approach). The lower and upper limits of the 95% CI (H_0_) are 59.9 and 70.2 respectively, with an expected mean of 60.3 for the experimental group. However, we may fail to detect a difference when actually there is a difference (i.e., the β-approach). Following similar differences between experimental and control groups in the Clemes et al. [17] study, this probability of failing to detect a difference when actually there is a difference is almost 50% (β = 0.54; power = 0.46).

### Intervention

In the experimental classroom, newly developed sit-to-stand desks replaced the regular desks. These sit-to-stand desks are called Adjust-Table Basic, and are manufactured and provided by Presikhaaf Schoolmeubelen, Arnhem, The Netherlands (https://www.schoolmeubelen.com). The desks have been designed to be easy to operate by young children. They are operated by a lever, which releases a gas spring lock. The gas spring allows very low-effort raising and lowering of the desktop. The desktops of the sit-to-stand desks have the same dimensions as the desktops of the regular desks (i.e., 500 × 700 × 18 mm). Height can be infinitely adjusted between 75 and 120 cm.

Each student in the experimental group received such a sit-to-stand desk, also the students that do not participate in the study. They will keep their sit-to-stand desks until they go to secondary school. The teacher of the experimental group also received a sit-to-stand desk (for adults). The students in the experimental group are not obligated to stand; rather, they are invited to stand, first by the mere opportunity to stand offered by the sit-to-stand desks (cf. affordance: the qualities or properties of an object that define its possible uses or make clear how it can or should be used), and second by the teacher who functions as a role model. For instructions, however, the teacher can order all children to sit for a proper view the (digital) blackboard at the front of the classroom. The students in the control group use their regular desks, as does their teacher.

### Primary outcome variables

Primary outcome measures are academic performance and the proportion of sitting time at school. Academic performance is assessed with the standardized and norm-referenced CITO test battery [43]. The standard procedure for most schools in The Netherlands is to assess academic performance twice each year with the CITO test battery. The scores of arithmetic, orthography, and reading comprehension are used in this study. Academic performance is assessed shortly before the week that sitting time is measured.

Sitting time is measured with an Activ8® Professional activity tracker [44, 45]. The Activ8® Professional (30 × 32 × 10 mm) is a 3D accelerometer that classifies postures and activities when worn on the upper leg: lying, sitting, standing, walking, cycling, and running. As with most other activity trackers, the Activ8® Professional is not able to validly identify all categories [28]. In youths, it can validly distinguish basic postures and activities (i.e., good to excellent validity), but has difficulty in distinguishing standing from other movements in complex activities; in complex activities, standing is often underestimated and detected as walking [44]. Each assessment (i.e., twice each year), an activity tracker is fixed on the upper leg of all participating students with a skin-friendly, waterproof, and transparent dressing (Tegaderm™, 3 M), halfway between the hip and knee. The activity tracker is worn for a school week during each assessment period: 24 h each day for five consecutive days from Monday morning till Friday afternoon. The recording interval is set at 10 s. At the end of the week data are collected via the Activ8® Professional recording tool.

### Secondary outcome variables

At each assessment period additional outcomes are measured following the week after sitting time is measured. Secondary outcomes are proportion of time spent in other postures and activities than sitting at school; proportion of sitting time and proportion of time spent in other postures and activities while awake outside school hours; cognitive skills; indicators of health; and indicators of wellbeing. Secondary outcome data, which may be influenced by the use of sit-to-stand desks [4, 19, 46–51], will be analyzed in relation to (changes in) sitting time.

The proportion of time spent in postures (i.e., lying, standing) and activities (i.e., walking, cycling and running) other than sitting at school are extracted from the Activ8® recordings. Additionally, to compare the proportion of time spent in postures and activities at school and outside school hours, the proportion of time spent in the different postures and activities outside school hours are also extracted. The times used for this are between 7:00 AM and 8:45 AM (the beginning of the school day), and between 3:00 PM (the end of the school day; 12:30 PM on Wednesdays) and 10:00 PM. Furthermore, the Activ8® recordings will also be used to compare posture and behavior with the wake up and sleep times recorded in the sleep diary.

Computer tests are used to study four dimensions of executive functioning that are related to and may underlie academic achievements: (I) working memory, (II) planning, (III) inhibition, and (IV) cognitive flexibility [52, 53]. All tests are presented using Inquisit 4 Computer Software [54] on a 15.6 in. ASUS N551 J 64-bit laptop computer screen. Completion of the four tests together takes between 30 to 60 min, dependent on the performance on each task. Children are administered the tests individually in a separate room under supervision of an assessor.

Handling working memory load is tested with the N-Back task [55, 56]. The N-Back task is a widely used working memory task that has face validity [57], although other types of validity, such as concurrent and convergent validity, are debatable [57–59]. In this modified version students are presented with a sequence of pictures (e.g., monkey, scissors, umbrella, chicken, cupcake), instead of letters. Pictures have been used in N-Back tasks before when testing with children [60, 61]. The task consists of indicating when the current picture matches the one from n steps before in the sequence. The load factor n is adjusted to make the task more or less difficult. In this study we use three levels: *n* = 1, *n* = 2, and *n* = 3. The first measure we use is the number of correct responses for each level. A high number of correct responses reflects a good handling of working memory load. The second measure is the mean response time for each level, which indicates the level of control over working memory processes [61, 62].

Planning is measured with the Tower of London task [63, 64]. This test starts with two pictures on the screen. Both pictures show a board with three vertical pegs of varying length, and three colored beads (i.e., red, blue and green). The first peg can hold three beads, the second two beads, and the third one bead. One picture shows the goal state and the other is the workspace board, where participants can rearrange the beads in the least number of moves from the start constellation to the goal state. There are twelve trials with increasing difficulty to complete. The first measure that is used is the total number of excess moves a participant makes beyond the minimum required moves. A low number of excess moves reflects good planning. The second measure of interest is the mean solution time. A low solution time reflects good efficiency on the task.

Inhibition is measured with an Inquisit version of the Fish Flanker test (FFT) [65]. The FFT is a basic derivative of the flanker visual filtering test that was developed by Eriksen and Eriksen [66]. The FFT is widely used to test one specific kind of response inhibition: the resistance to distractor interference. This refers to the ability to efficiently ignore irrelevant visual distractor information while processing target stimuli. A trial consists of a picture with five identical fishes. One fish in the center and two fishes on each side of the center fish. The direction the center fish is facing (i.e., direction of the head of the fish) determines what button should be pressed, left or right. All neighboring fishes move in the same direction as the center fish in congruent trials, or in the opposite direction in incongruent trials. Trials are interpreted as false in three cases: (I) when the wrong button is pressed; (II) when the response time to a trial exceeds 3000 milliseconds (an inattentive error); or (III) when the response time is shorter than 200 milliseconds (early response). The measures of interest are the number of erroneous responses and the mean response time, each registered for both the congruent and the incongruent trials. Both a low error rate and a low response time reflect good resistance to distractor interference.

Cognitive flexibility is measured with a digital version of the Wisconsin Card Sorting Task [67]. In this task a student is presented with cards that vary in the presented shapes on them, the color of the shapes, and the number of shapes. The student is to match the upper “example” card on the screen with one of four cards presented below the example card by clicking with the computer mouse on one of them. The sorting rule in effect is the dimension to which the correct choice matches the example card, and the student should find out what sorting rule is in effect. By using the feedback given to the student when making a correct or a false choice the student can theoretically find the sorting rule in effect after two trials. There are three categories, each containing four different values: (I) color, (II) form, and (III) number. Each category is presented twice. In our modified version, the criterion for successful completion of a category is applying the correct sorting rule four times in succession. After a streak of four correct responses, the sorting rule changes without the student being informed that it will. The maximum number of trials is set at 128. The measure of interest is the number of perseverative errors. This is the number of errors where the student continues to sort cards according to the same rule despite negative feedback. A low number of perseverative errors reflects good cognitive flexibility.

Indicators of health are measured with a number of tests and diaries. The 20-m Shuttle Run Test (SRT) is used to measure physical fitness [68]. The SRT is a widely used, and validated test [69]. It is a simple, easy to administer test, and a large number of individuals can be tested simultaneously. The SRT test consists of stages of continuous, incremental speed running between two lines 20 m apart. The initial speed is set at 8.5 km/h and increases by 0.5 km/h per minute. Audio signals indicate the speed. The test ends when a student fails to reach the end lines concurrent with the audio signals on two consecutive occasions. The reached stage is recorded. The assessment is carried out by an assessors together with the physical exercise teacher.

Hand dynamometry is used to measure grip strength. A hand dynamometer is a valid and reliable tool for measuring upper body strength and hand function [70]. Each student is tested with a calibrated Jamar® hydraulic dynamometer (J.A. Preston Corporation, Clifton, NJ). The dynamometer is set at the second handle position, and the standardized testing position for measuring grip strength is used [71]. The students are allowed a total of four attempts; twice with each hand. The highest score (in kg) for each hand is recorded by the assessor.

Lower body power is assessed by the assessor with the vertical jump, as first described by Sargent in 1921 [72]. The vertical jump is a practical, low-cost, reliable [73], and valid [74] test for two-legged explosive power. First the standing reach height is measured: the student stands straight beside a wall and reaches up with the hand closest to the wall, while keeping the feet flat on the ground. The point of the fingertips is recorded using a wall mounted measuring tape (Seca 206; Seca gmbh & co. kg., Hamburg, Germany). Then the student jumps vertically from a flat footed position and tries to touch the wall as high as she or he can. The highest point of three attempts is recorded. The distance to the nearest 0.5 cm between the standing reach height and the highest vertical jump height is the recorded score.

Each student’s weight (kg) and height (cm) is measured with light clothes and without shoes by the assessor. Weight is measured to the nearest 0.1 kg with a validated digital scale (Omron BF511; Omron Corp, Kyoto, Japan) [54]. Height is measured to the nearest 0.5 cm using a wall mounted measuring tape (Seca 206; Seca gmbh & co. kg., Hamburg, Germany). When measuring height, the student stands on a level floor with heels together; weight evenly distributed; heels, buttocks and shoulders against the wall; and arms loosely hanging at the sides with palms facing the thighs. BMI is calculated using the following formula: body mass/height^2^ (kg/m^2^). The scale used to measure body weight also allows bioelectrical impedance analysis of the body composition [75]. The student stands barefoot on metal footpads and grasps a handle with arms extended in front of her/his chest. Percentages of total body fat and total body muscle are measured with eight electrodes (two under each foot and two in each hand) and recorded.

A diary including the Dutch version of the Bristol Stool Form Scale for children (BSFSC) is used to report stool [76]. The BSFSC is a valid and reliable method for children to keep record of their stool [77]. At the start of the same week that the students wear their activity tracker, they receive their BSFSC. For five consecutive school days students write down their stool form (five categories ranging from “separate hard lumps like nuts” to “watery, no solid pieces), stool frequency, occasions of abdominal pain and pain during defecation, and whether they use medication for their stool.

In the same week that students keep a stool diary, they also keep a sleep diary. The diary is based on the Consensus Sleep Diary [78], which has become the de-facto sleep diary in sleep research, but has not yet been validated for children. With the sleep diary students keep track of the time they go to bed, fall asleep at night, get out of bed in the morning, and how often they wake up at night. They also rate their sleep and how well-rested they are, and report whether medication is needed to sleep. This diary is also used to confirm two transitions between inactivity and activity (i.e., the times of falling asleep and waking up) as recorded with the activity tracker. Parents are asked to remind the students to fill in both diaries.

Indicators of wellbeing are measured with questionnaires, which are assessed in the classroom under supervision of the teacher. A faces scale is used to measure happiness [79, 80]. The student answers to questions by indicating one face of a five-points-smiley-face-scale ranging from a green, happy face to a red, sad face. All six questions address the student’s feeling of happiness in general or in a specific situation (e.g., “How do you feel at the moment?” and “How do you usually feel at school?”). This questionnaire is not validated.

The KIDSCREEN-52 is a questionnaire to measure quality of life (QoL) in children and adolescents [81]. This is a reliable and valid, generic instrument that is used throughout many countries in the world [82]. The KIDSCREEN questionnaire consists of 52 items to be answered by the student. Students’ QoL is assessed in ten dimensions: physical well-being; psychological well-being; moods and emotions; self-perception; autonomy; relations with parents and home life; social support and peers; school environment; social acceptance (bullying); and financial resources. The items are five-point Likert scales to assess either the frequency (never to always) of certain behaviors/feelings or the intensity of an attitude (not at all to extremely). The recall period for each item is 1 week.

Satisfaction with the school environment is assessed with a short questionnaire. Eight items address the students’ satisfaction with the school building, their classroom, and their school furniture (e.g., “I am satisfied with how our classroom looks.” and “The furniture in our classroom is looking good.”). Students rate to what extent they agree or disagree on a four-point Likert-scale. These eight items are selected from the Dutch Quality Indicator in Primary Education (Kwaliteitsmeter Primair Onderwijs; Van Beekveld & Terpstra, Hoorn, The Netherlands); a commonly used, however non-validated, student questionnaire to rate the quality of a school.

Records of adherence and adverse events are also kept. Adherence is defined as the percentage of students in the experimental group who keep participating in the study measured at every test week. Adverse events are defined as any undesirable experience occurring to a subject during the study, whether or not considered related to the investigational product, testing procedures or experimental intervention. All adverse events reported spontaneously by the student or observed by a teacher, parent or assessor are recorded.

A complete list of primary and secondary outcome measures and supervision is provided as Additional file 2. Regarding supervision, assessors are present during computer testing, SRT, hand dynamometry, vertical jump, and height, weight, and body composition assessments. Teachers are present when students fill out questionnaires, and parents help the students to keep record of diaries.

### Data handling

Data are handled confidentially and anonymously. Each student’s data are stored under a unique eight-digit code that is not related to the student’s name, initials or birth date. The key to the codes remains at the school with the director. All research data (i.e., data with codes) are stored on a secured server computer at the Leyden Academy. The school (code key) and the Leyden Academy (data) are physically separated. The handling of data complies with the General Data Protection Regulation [83]. The data will be stored for 10 years. In all reporting no codes will be reported.

The database will be managed in Microsoft Excel 2016 and IBM SPSS Statistics, Version 23.0. The results of the Activ8® Professional activity tracker are collected with the Activ8® Professional recording tool; the computer test data from the executive functioning tests are first collected with the Inquisit 4 Computer Software. Data collected on paper forms are checked and data entry is performed by the Data Manager at Leyden Academy. All authors (AEQ, GPH, JPJ) are given access to the full dataset.

### Statistical analysis

The statistical analysis will be conducted in SPSS. A two-tailed significance level of .05 will be used for all tests.

Summary statistics for the baseline measures and participant demographics for both groups will first be examined. Dependent on the variable, the means, medians or frequencies will be compared between both groups using a two-sample T-test, a Mann-Whitney U test or chi-square test, as appropriate.

Outcome data obtained from all students will be included in the data analysis, and data will be analyzed as per group allocation. In our statistical analysis the longitudinal and multilevel structure of the data are acknowledged and will be taken into account in achievement growth modeling. For this, mixed modeling will be used, because this specifically allows for the analysis of repeated measures and nested data. Furthermore, missing data are better handled in mixed modeling. We will first determine the growth (longitudinal changes) for the different outcome measures on an individual and on a group level. In determining the proportion of variance explained by group allocation (i.e., sit-to-stand or regular desks) for sitting time as well as for academic performance (i.e., the primary outcome measures), explanatory variables such as age, gender and baseline outcomes will be added to the model. We will do this both on an individual and on a group level, also for a number of secondary outcome measures. We will test for significance of the effect of group allocation on a group level.

## Discussion

Sedentary behavior is associated with academic under-achievement and health risks in children [4, 5]. Still, children spend a large part of their waking hours sitting at a desk at school [6]. The program of “A Good Beginning” was conceived to assess the long-term effects of sit-to-stand desks on academic performance and sitting time in primary education, and to examine how sit-to-stand desks versus regular desks relate to measures of executive functioning, health and wellbeing. The paper presents the design of this group-randomized trial.

The program of “A Good Beginning” is expected to provide a significant contribution to the understanding of the effects of sit-to-stand desks on sedentary time at school and academic performance. The program is contingent upon the fact that stakeholders learn to understand the detrimental effects of long-term inactivity and excessive sedentary behavior, also at a young age. Given that children spend a large part of their waking hours at school sitting at a desk [6], and the fact that the environment has a very strong influence on behavior [13–15], the school setting, in particular, is the place to reduce children’s sedentary time. Accordingly, this study is relevant and needed to objectively guide policy making and practical decision making with regard to school and classroom environments.

Recent short-term studies have demonstrated relevant benefits of using sit-to-stand desks with regard to sitting time reduction [16–20], without negatively affecting academic performance [26]. Moreover, in their study with grade one students (6 to 7 years old), Blake et al. found a positive effect of the use of desks that promote standing on attention and focus, as reported by the teachers [24]. In the study by Dornhecker et al. second to fourth grade students were observed by trained research assistants [26]. Based on these observations, they conclude that students with desks that promote standing exhibited greater levels of academic engagement (i.e., activities such as answering a question, raising a hand, participating in active discussion) than students with regular desks. However, Koepp et al. did not find a significant difference in teacher reported concentration between sixth graders that used desks that promote standing and those that used regular desks [31]. The program of “A Good Beginning” is a unique project that goes beyond the short-term view by covering two-and-a-half years, with assessments twice a year in winter and summer seasons. Consequently, it will not suffer from effects of novelty, and effects of season are taken into account. The program specifically allows insight into the combination of assessments of objective sedentary time and academic performance over a longer period of time, which is of particular interest to educators.

The study also knows some limitations. First, the sample size is most likely too small for detecting a significant difference in sitting time. The downside is that a small sample size limits population estimation and extrapolation of findings. In addition, a possible true effect may be masked by a relatively large variance in our sample (this is the reason why we calculated the probability of failing to detect a difference). On the upside, in the study we use a repeated-measures design instead of the pre-post design that was used in the study by Clemes et al. [17]. This increases the power to some extent. However, given the fact that the primary aim was to assess possible harm to academic performance, the effect of sit-to-stand desks on sedentary time reduction (and the related sample size calculation based on results the study by Clemes et al. [17]) was considered subordinate to the investigation of possible harm to academic performance (and the related non-inferiority calculation). Given the circumstances, we therefore accepted a small sample size, which is around half the sample size of the study by Clemes et al. [17], and we will treat the outcomes with cause.

An additional limitation follows from the group randomization, which is inevitable in studies with school classes. Group randomization has a negative effect on the power of the study. With the proposed statistical analysis (i.e., mixed modeling), we will be able to determine the extent of the negative effect of this design on the power of the study and discuss the consequences.

Furthermore, this study involves only one school. Potential limited external validity and problems with blinding are common limitations in single-center (ic. single-school) studies, which may also apply to this study. We will take these limitations into consideration in our analyses and findings.

The program of “A Good Beginning” started in 2017 and results are expected by the beginning of 2020. The results of this study may provide relevant information to set up a larger scale efficacy trial, possibly with children of different ages (also in secondary education), more schools with different populations, and a protocol focusing primarily on sedentary time reduction.

## Supplementary information

**Additional file 1.** SPIRIT Checklist.

**Additional file 2.** Outcome Measures.

## Data Availability

The study is ongoing. The datasets that will be used and/or analysed during the study will be available from the corresponding author on reasonable request.

## Abbreviations

BSFSC: Bristol Stool Form Scale for Children
CCMO: Dutch Central Committee on Research Involving Human Subjects (Centrale Commissie Mensgebonden Onderzoek)
CITO: Central Institute for Test Development (Centraal Instituut voor Toets Ontwikkeling)
FFT: Fish Flanker Test
QoL: Quality of Life
SRT: 20-m Shuttle Run Test
WMO: Medical Research Involving Human Subjects Act
