# Supplementary material for: A good beginning: study protocol for a group-randomized trial to investigate the effects of sit-to-stand desks on academic performance and sedentary time in primary education
Source: BMC Public Health. 2020 Jan 15;20:70. doi: 10.1186/s12889-019-8135-9 (PMC6964001; doi:10.1186/s12889-019-8135-9)
Supplement: Supplementary file 1 — Additional file 1. SPIRIT Checklist. [file 12889_2019_8135_MOESM1_ESM.docx]

**Additional File 1**

**SPIRIT Checklist for “A Good Beginning”**

**Administrative information**

1. Title: Page 1, Line 3
2. Trial registration:
   1. Registry: Page 3, Lines 53-55, Trial Registration
   2. Data set: Page 38, Line 913, WHO Trial Registration data Set
3. Protocol version: No amendments to the study protocol have been made. The study protocol has received ethical approval from the Dutch Central Committee on Research Involving Human Subjects (CCMO, https://english.ccmo.nl, number NL60159.000.17; see also the manuscript Page 19, Lines 452-455). However, there were three “question rounds” in the correspondence with the CCMO, resulting in three “versions” of the protocol (which are stored both at the CCMO and on the secured server at Leyden Academy on Vitality and Ageing). See also the document in Dutch regarding ethical approval: A Good Beginning Ethical Approval 2017. The following is a translation of the text of the 2^nd^ paragraph on the 4^th^ Page, starting from the second Line: “The most important questions [*by the CCMO*] addressed (I) the effect of physical activity stimulation in the proposed age range, (II) the question if children who could not stand for short period of time (for example due to sports injury) would still be able to participate in the study, and (III) the explanation for the length of the study (time to follow-up). Additionally, an explanation of the power calculation was requested, as well as a reason for recruiting the small number of participants [*i.e. students from only two groups*] and possible withdrawal. In reaction to these questions, an new version of the protocol was sent together with [*by the applying researchers*] additional documents. After having received the this information, it was concluded that all questions have been addressed satisfactorily.” In conclusion, there is no remark in the manuscript of any version or change of the protocol; the only information that is available on protocol “versions” can be found in the correspondence with the CCMO, which (is in Dutch, and) has been sent before under A Good Beginning Ethical Approval 2017.
4. Funding: Page 26, Line 624, Declarations, Funding (the Municipality of Leiden provided a grant of €45K; see also the document A Good Beginning Grant Leiden Municipality 2017 2018 2019; also in Dutch). Page 11, Lines 247-248, Methods, Intervention, first paragraph; Page 26, Lines 624-625, Declarations, Funding (Presikhaaf Schoolmeubelen provided the sit-to-stand desks to the school; they are now school property).
5. Roles and responsibilities:
   1. Contributorship: Page 26, Lines 637-643, Declarations, Authors’ Contributions.
   2. Sponsor contact information: Page 1, Line 9 and Lines 20-23, Corresponding Author.
   3. Sponsor and funder: Page 26, Line 624-627, Declarations, Funding.
   4. N/A.

**Introduction**

1. Background and rationale:
   1. Background and rationale: Pages 4-6, Lines 76-144, Background (complete).
   2. Choice of comparators: Page 5, Lines 107-115, Background, second paragraph; Page 11, Lines 246-253, Methods, Intervention, first paragraph; Page 11, Lines 255-264, Methods, Intervention, second paragraph.
2. Objectives: Page 5, Lines 117-124, Background, fifth paragraph; Page 6, Lines 126-134, Background, sixth paragraph; Pages 21-22, Lines 507-540, Discussion, paragraphs 1-3.
3. Trial design: Page 5, Lines 107-110, Background, fourth paragraph; Page 7, Lines 157-165, Methods, Design, first paragraph.

**Methods: Participants, interventions, outcomes**

1. Study setting: Page 8, Lines 185-188, Methods, Recruitment.
2. Eligibility criteria: Page 8-9, Lines 193-200, Methods, Recruitment.
3. Interventions:
   1. Interventions: Page 11, Lines 246-264, Methods, Intervention, paragraphs 1-2.
   2. Modification: N/A.
   3. Adherence: Page 11, Lines 258-261, Methods, Intervention, second paragraph. Note that this is actually not an adherence strategy but simply part of the intervention. The use of the different functions of the sit-to-stand desks is at the student’s ow disposal. Page 19, Lines 454-456, Methods, Secondary outcome variables, paragraph 17.
   4. Concomitant care: Page 11, Lines 263-264, Methods, Intervention, second paragraph. In fact, the students in the control group receive “usual care”.
4. Outcomes: Pages 11-12, Lines 267-286, Methods, Primary outcome variables, paragraphs 1-2 (for primary outcome measures); Pages 12-19, Lines 288-460, Methods, Secondary outcome variables, paragraphs 2-17.
5. Participant timeline: Page 7, Lines 157-161, Methods, Design, first paragraph, and Figure 1 (Page 7, Line 167, Methods, Design).
6. Sample size: Page 9-10, Lines 212-243, Methods, Sample size, paragraphs 1-3.
7. Recruitment: Page 8-9, Lines 185-207, Methods, Recruitment, and Table 1 (Page 9, Line 208).

**Methods: Assignment of interventions (for controlled trials)**

1. Allocation:
   1. Sequence allocation: Page 7, Lines 161-163, Methods, Design, first paragraph; Page 8, Lines 188-191, Methods, Recruitment, first paragraph; Figure 1 (Page 7, Line 167, Methods, Design).
   2. Concealment mechanism: Page 8, Line 190-191, Methods, Recruitment, first paragraph; Figure 1 (Page 7, Line 167, Methods, Design).
   3. Implementation: Page 7, Lines 161-163, Methods, Recruitment, first paragraph; Pages 7, Lines 169-173, Methods, Recruitment, second paragraph; Page 8, Lines 188-191, Methods, Design, first paragraph; Figure 1 (Page 7, Line 167, Methods, Design).
2. Blinding:
   1. Blinding (masking): Pages 7, Lines 169-173, Methods, Recruitment, second paragraph.
   2. Emergency unmasking: N/A.

**Methods: Data collection, management, analysis**

1. Data collection methods:
   1. Data collection methods: Pages 11-12, Lines 267-286, Methods, Primary outcome variables, paragraphs 1-2 (for primary outcome measures); Pages 12-19, Lines 290-460, Methods, Secondary outcome variables, paragraphs 2-17.
   2. Retention: No specific strategies for retention are implemented. We will, however, ask for the reason(s) why in case a student terminates its participation in the study (Page 9, Lines 204-205, Methods, Recruitment, first paragraph).
2. Data management: Pages 20, Lines 469-482, Methods, Data handling, paragraphs 1-2.
3. Statistical methods:
   1. Outcomes: Pages 20-21, Lines 485-504, Methods, Statistical analysis, paragraphs 1-3.
   2. Additional analyses: N/A.
   3. Analysis population and missing data: Page 21, Line 497, Methods, Statistical analysis, third paragraph.

**Methods: Monitoring**

1. Data monitoring:
   1. Formal committee: N/A.
   2. Interim Analysis: N/A.
2. Harms: Page 7, Lines 151-153, Methods, first paragraph.
3. Auditing: N/A.

**Ethics and dissemination**

1. Research ethics approval: Page 24, Lines 586-588, Declarations, Ethics approval and consent to participate, first paragraph.
2. Protocol amendments: N/A.
3. Consent or assent:
   1. Consent or assent: Pages 24-25, Lines 590-611, Declarations, Ethics approval and consent to participate, paragraphs 2-3.
   2. Ancillary studies: N/A.
4. Confidentiality: Page 20, Lines 469-475, Methods, Data handling, first paragraph.
5. Declaration of Interests: Page 26, Line 621, Declarations, Competing interests.
6. Access to data: Page 20, Lines 481-482, Methods, Data handling, second paragraph.
7. Ancillary and post-trial care: Page 11, Lines 256-257, Methods, Intervention, second paragraph. Given the nature of the study and products used in the study, no further ancillary and post-trial care will be provided other than would normally be provided to school children. Obviously, the sponsor has a liability insurance which is in accordance with Dutch legislation, and an insurance that provides cover for damage to research subjects through injury or death caused by the study. The insurance applies to the damage that becomes apparent during the study or within 4 years after the end of the study. This information is available at the CCMO and Leyden Academy.
8. Dissemination policy:
   1. Trial results: Page 26, Lines 630-635, Declarations, Public disclosure and publication policy.
   2. Authorship: Pages 26-27, Lines 638-643, Declarations, Authors’ contributions.
   3. Reproduceable research: Page 26, Lines 617-618, Declarations, Availability of data and materials, and Lines 630-635, Declarations, Public disclosure and publication policy. Furthermore, information about the study protocol is publicly available at the Netherlands Trial Register and the CCMO-register.

**Appendices**

1. Informed consent materials: Page 8, Line 190-191, Methods, Recruitment, first paragraph; Page 24, Lines 586-588, Declarations, Ethics approval and consent to participate, first paragraph; Page 25, Lines 596-599, Declarations, Ethics approval and consent to participate, second paragraph.
2. Biological specimens: N/A.
