# Supplementary material for: A good beginning: study protocol for a group-randomized trial to investigate the effects of sit-to-stand desks on academic performance and sedentary time in primary education
Source: BMC Public Health. 2020 Jan 15;20:70. doi: 10.1186/s12889-019-8135-9 (PMC6964001; doi:10.1186/s12889-019-8135-9)
Supplement: Supplementary file 2 — Additional file 2. Outcome Measures. [file 12889_2019_8135_MOESM2_ESM.docx]

Additional file 2

| **OUTCOME MEASURES** |  |  |  |  |
| --- | --- | --- | --- | --- |
|  |  |  |  |  |
| **Primary outcome measures** | | Instrument | Supervision |  |
| Academic performance | Arithmetic | CITO | Teacher |  |
|  | Orthography | CITO | Teacher |  |
|  | Reading comprehension | CITO | Teacher |  |
| Postures and activities | Proportion sitting time at school | Activ8 |  |  |
|  |  |  |  |  |
|  |  |  |  |  |
| **Secondary outcome measures** | | Instrument | Supervision |  |
| Postures and activities | Proportion lying time at school | Activ8 |  |  |
|  | Proportion standing time at school | Activ8 |  |  |
|  | Proportion walking time at school | Activ8 |  |  |
|  | Proportion cycling time at school | Activ8 |  |  |
|  | Proportion running time at school | Activ8 |  |  |
|  | Proportion lying time outside school | Activ8 |  |  |
|  | Proportion sitting time outside school | Activ8 |  |  |
|  | Proportion standing time outside school | Activ8 |  |  |
|  | Proportion walking time outside school | Activ8 |  |  |
|  | Proportion cycling time outside school | Activ8 |  |  |
|  | Proportion running time outside school | Activ8 |  |  |
| Cognitive skills | N-Back task (working memory) | Inquisit 4 | Assessor |  |
|  | Tower of London (planning) | Inquisit 4 | Assessor |  |
|  | Fish Flanker Test (response inhibition) | Inquisit 4 | Assessor |  |
|  | Wisconsin Card Sorting Task (cognitive flexibility) | Inquisit 4 | Assessor |  |
| Indicators of health | Shuttle Run Test (physical fitness) |  | Assessor |  |
|  | Hand dynamometry (grip strength) | Jamar | Assessor |  |
|  | Vertical jump (lower body power) | Seca 206 | Assessor |  |
|  | Weight | Omron BF511 | Assessor |  |
|  | Height | Seca 206 | Assessor |  |
|  | Body Mass Index | Omron BF511 | Assessor |  |
|  | Total body fat | Omron BF511 | Assessor |  |
|  | Total body muscle | Omron BF511 | Assessor |  |
|  | Stool | BSFSC | Parent |  |
|  | Sleep | Consensus Sleep Diary | Parent |  |
| Indicators of wellbeing | Happiness | Faces scale | Teacher |  |
|  | Quality of Life | KIDSCREEN-52 | Teacher |  |
|  | Satisfaction with school environment | Selection of questions | Teacher |  |
| Extra | Adherence |  |  |  |
|  | Adverse events |  |  |  |
